# A Badge Design Framework for a Gamified Learning Environment: Cases Analysis and Literature Review for Badge Design

**DOI:** 10.2196/14342

**Published:** 2019-05-29

**Authors:** Sungjin Park, Sangkyun Kim

**Affiliations:** 1 Management of Technology Lab Kangwon National University Chuncheon Republic of Korea

**Keywords:** badge design framework, educational badge, digital badge, badge types, gamified learning environment

## Abstract

**Background:**

In the past, the educational badge was an extrinsic means of rewarding the motivation to learn. Based on continued research, however, the badge began to be recognized as a scale to measure the learner’s knowledge and skill and an important means of helping learners to gradually build intrinsic motivation by using certain extrinsic motivators. As the badge’s value has grown, the importance of its design has garnered attention.

**Objective:**

The objective of this research was to establish a badge design framework that can be used in a gamified learning environment.

**Methods:**

Data were collected from previous studies on badge design, 943 badge cases were extracted from 11 online and offline gamification in education contents, and their patterns and features were analyzed.

**Results:**

Based on the analysis of results from previous studies and 943 collected badge cases, our study suggests three conditions for badge design. Through the literature review and collected badge cases, our study designed a badge design framework. First, it is necessary to distinguish whether the type of learning activity required for earning badges is physical or conceptual. Second, it is necessary to distinguish whether the scale of an activity required for earning badges requires individual learning or interaction-induced learning. Third, it is important to review whether the time and effort invested in earning badges is simple, repetitive, and short-term or continuous and long-term. Based on these three conditions, collected badge cases were analyzed. To verify self-developed badge types, we conducted a chi-square test on the collected cases and confirmed that there was a significant difference for each of the eight badge types (Pearson chi-square 1117.7, *P*<.001).

**Conclusions:**

Through its literature review on previous studies, this study demonstrated the badge’s educational effectiveness. The badge design framework suggested in our study is expected to resolve some of the difficulties experienced during the badge design process in a gamified learning environment, encourage efficient badge design, and maximize learning effect.

## Introduction

### Gamification and Digital Badges

**Figure 1:**
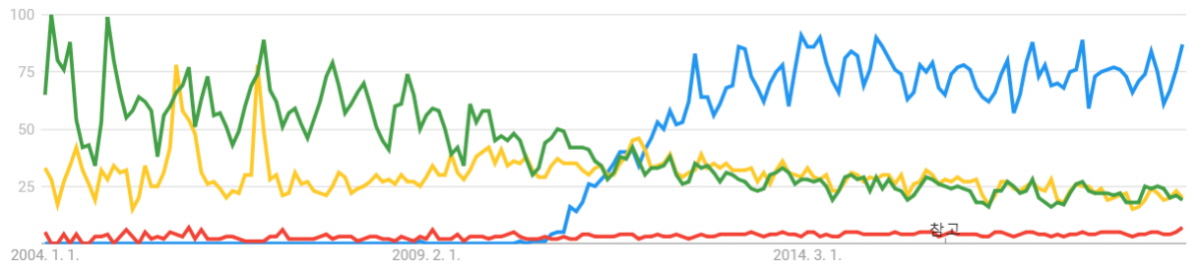
Keyword search results in Google Trend (blue: gamification, red: game-based learning, yellow: serious game, green: educational game). The x-axis is time from 2004 to April 11, 2019. The y-axis is the search volume provided by Google Trends and range is 0 to 100.

**Figure 2:**
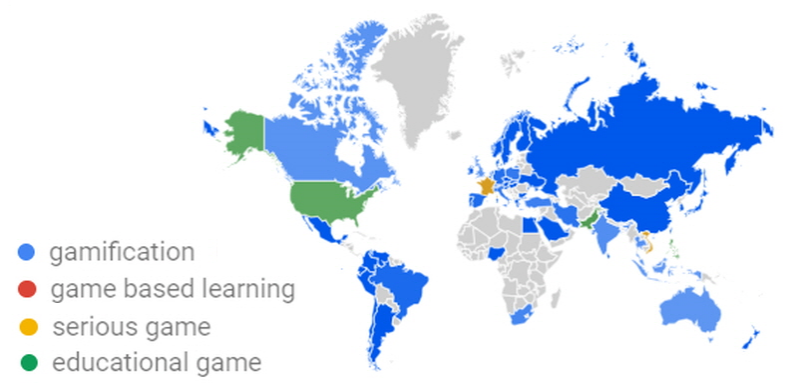
Regional interest by country in Google Trends from 2004 to April 11, 2019 (deeper color indicates more interest).

Gamification in education applies game elements to an educational context [1]. Through game mechanics such as badges, leaderboards, and avatars, feedback is provided to the learner and encourages collaboration and cooperation [2,3]. In the education context, gamification has been touted to overcome the shortcomings of traditional learning methods; it has been claimed that it could offer learners new experiences and values [4]. Before gamification, educational games, game-based learning, and serious games were applied to the classroom. Since gamification was defined, however, it has become preferred among instructors compared with the other techniques (Figures 1 and 2). Al-Azawi et al [5] compared gamification, game-based learning, and educational gaming. We analyzed the benefits of gamification in these same contexts. The contexts are provided below along with their respective advantages:

- Gamification in education: better learning experience, better learning environment, instant feedback, better prompting of behavioral change, and better applicability in terms of most learning needs compared with game-based learning and educational games
- Game-based learning: increases learner’s memory capacity and computer and simulation fluency, helps to quicken strategic thinking and problem solving, develops hand-eye coordination, and facilitates skill-building
- Educational gaming: improves motor skills, social development, focus and memory ability, self-esteem, and creativity

**Figure 3:**
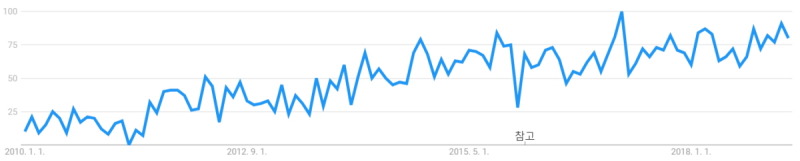
Interest in digital badges as shown in Google Trends. The x-axis is time from 2004 to April 11, 2019. The y-axis is the search volume provided by Google Trends and range is 0 to 100.

**Figure 4:**
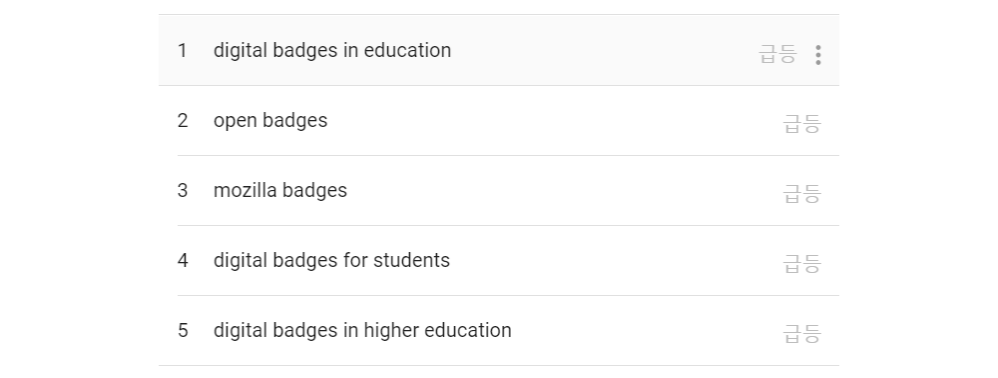
Google Trends keyword search result related to digital badges.

As interest in online learning environments has grown greatly, so has interest in digital badges. We conducted a keyword search for digital badges in Google Trends. The results showed that its search trend has been on the rise since 2010 (Figure 3); furthermore, terms related to the education context were found to be related search words (Figure 4). Badges, which were once used as mere extrinsic rewards, were actively used in the gamified learning environment.

The badge is a product of the learner’s invested time and efforts; furthermore, it functions as a scale that indirectly indicates one’s ability level to others [6]. It can be applied in both online and offline education environments. From a pedagogical viewpoint, the use of badges can help to introduce innovation to the education environment and thus have a positive effect on promoting learning achievements [7]. For this reason, the process of designing a badge is important. Most badges are designed based on the experiential judgment of the designer, teacher, or decision maker. Designing a badge based on the relevant theoretical background, evidence from previous cases, and the designer’s experience, however, makes the badge more efficient for use. Therefore, in this study we looked into previous research, collected 943 badges from 11 online and offline educational sources, and analyzed their patterns and features in order to determine an efficient badge design.

### Background and Literature Review

Al-Azawi et al [5] point out the differences between gamification and the two previously mentioned techniques of game-based learning and educational games in terms of the implementation method, cost, and applicability. While gamification is affordable in terms of development and easier to implement, the teacher may find it hard to access the other two techniques of game-based learning and educational games, since they should be developed like actual video games using computer or console game such as PlayStation (Sony Interactive Entertainment LLC) or Xbox (Microsoft Corp). In addition, these techniques tend to be expensive in terms of development.

It was suggested that gamification in education can deliver a gameful experience in the education environment through game design elements, facilitate the learner’s use of game-like thinking and strategies, and provide an immersive learning experience. De Sousa Borges et al [8] conducted a systematic mapping review of 357 previous studies related to gamification in education. The review established seven categories that could be used to analyze such studies. Based on the results, the review suggested that the learning environment could be improved and learning performance enhanced through the following seven features in gamification in education:

- Mastering skills: enhance or improve the learner’s ability through complex and repeated activities that use gamification in education
- Challenge: aid the learner in actively participating in learning activities to improve their learning
- Guidelines: provide the theoretical background that helps with gamification settings in the education context
- Engagement: maintain or promote the learner’s interests in learning activities
- Learning improvement: reinforce the learner’s learning activities through a gamified solution and maximize the outcomes of the learning process
- Behavioral change: encourage and facilitate changes in the learner’s behaviors through the gamified system
- Socialization: provide an efficient learning behavioral change through the gamification for social activities such as communication and decision-making

Gamification in education contexts can also induce affordance in terms of learning. Majuri et al [9] conducted an empirical study on previous studies related to gamification in education. Out of 807 previous studies related to the research topic, the study selected 128. Analysis of these 128 previous studies showed that gamification in education could induce affordance; have a significant effect on psychological factors such as improved cognitive function, immersion, fun, and engagement; and change and encourage behaviors.

Gamification in education helped to introduce innovation into the education environment by providing many benefits to the learner. As gamification began to be applied into the education environment, many different game mechanics began to be used. Dicheva et al [10] carried out a systematic mapping review of previous gamification in education studies that had been conducted between 2010 and 2014. This review revealed that the highest number of previous studies on gamification in education had been conducted in 2013; in these studies, gamification was used to indicate learning status and improve social engagement. Furthermore, they found that the game mechanics were most used in the order of badge, leaderboard, point, level, virtual goods, and avatars. Of the 754 gamification cases analyzed by Park and Kim [11], 127 were related to gamification in education. In 73 of these cases (57.5%), badges were applied to learning behavior. Recently, gamification has begun to be applied to online learning platforms, and studies are being actively conducted on the digital badges applicable to the online learning environment. Gibson et al [6] stated that when digital badges are used in the education environment, it is possible to establish an affordance-based learning environment, motivate learning through the use of badges, and self-check one’s learning status; thus, affordance is expected to work to encourage the learner to reach their goals by representing invisible learning achievements as visible ones. McIlvenny [12] suggested that information for such badges should include badge icon, issuer, issue data, badge details, badge criteria, and evidence when disclosing the badges. Based on five identifiable items out of these six, we collected badge cases.

## Methods

### Data Collection

To collect badge cases, Google search was used and previous studies and related books were reviewed. The cases were collected from Dec 1, 2018, to January 31, 2019. The search keywords were badge in gamification, badge example in gamified learning (classroom), educational badges, and (gamification in education contents names) badges examples. The collected cases were summarized and arranged in Excel 2013 (Microsoft Corp). The study referred to six types of digital badge metadata that were suggested by McIlvenny [12] for badge information.

To make a badge design framework, we considered the mutually exclusive collectively exhaustive (MECE) approach. Compared with previous studies, we have minimized the weaknesses of self-developed badge design framework considering MECE. To verify a self-developed badge design framework, we conducted a Pearson chi-square test using SPSS Statistics version 23 (IBM Corp).

To present an efficient badge design framework, we collected 943 badge cases from 11 online and offline gamification in education contents that used badges and were available to the public. Table 1 provides information on the collected badges.

Codecademy [13], Codecombat [14], Khan Academy [20], and Sololearn [25] are gamification in education platforms that provide contents related to computer language learning. Duolingo [15] is a gamification in education platform for language learning. Codecademy (forum) [13] and Memrise (forum) [21] are online community platforms that were created for sharing user’s opinions; they use badges to encourage and promote activities in their communities. Edge [18] is a gamified customer relationship management system that manages customer loyalty based on the point, badge, and leaderboard/level system [26]. Fitbit [19] and Nike Plus [22] manage the amount of exercise accomplished by users based on mobile apps and hardware. Pokemon Go [24] is a Global Positioning System–based mobile augmented reality (AR) activity app. While it was approached as a game in its early days, Althoff et al [27] recognized Pokemon Go as gamified content, since it positively contributes to increasing physical activities. Thus, it was included as one of the cases in this study. Passport to Success [23] is a badge that is used for improving learning motivation and behavioral changes in schools located in the Corona-Norco Unified School District, California, United States.

In these cases, the time periods required to earn badges were calculated in terms of the number of days. The time periods used in the cases, on a minute or hour basis, were converted into decimals based on a period of 24 hours (one day). In the collected cases, the unit used to measure physical activities, such as walking, running, and walking upstairs, was converted into kilometer. We referred to the Kyle’s Converter website [28] in order to convert the number of steps.

**Table 1:** Introduction of gamification in education for badge collecting.

Gamified learning contents	Category	Type	Badges, n	Reference
Codecademy (forum)	Community	Software (online)	60	[13]
Codecombat	Education	Software (online)	51	[14]
Duolingo	Education	Software (online)	44	[15-17]
Edge	Royalty management	Software (online)	24	[18]
Fitbit	Health care	Software (app + hardware)	62	[19]
Khan Academy	Education	Software (online)	97	[20]
Memrise (forum)	Community	Software (online)	50	[21]
Nike Plus	Health care	Software (app + hardware)	78	[22]
Passport to Success	Education	Print out work + software (online)	300	[23]
Pokemon Go	Health care	Software (app)	123	[24]
Sololearn	Education	Software (online)	54	[25]

The numerical values for the other activities, except the physical ones (eg, solving quizzes, earning likes, and so on), in the collected badge cases were input without conversion:

- Badge name: name of the badge used in learning content
- Application domain: domain of the learning content that applies to a badge
- Reward criteria: activity criteria required for earning a badge
- Activity interval: period of time spent in earning the badge. Recorded on the basis of a day (24 hours) (eg, 5 hours = 0.21 days, 3 weeks = 21 days, 1 year = 365 days)
- Activity amount-1: physical activities (walking, running, and walking upstairs), among others, conducted to earn the badge. Converted into km (eg, 1 mile = 1.61 km, 1 step and 1 stair step = 0.0008 km)
- Activity amount-2: quantitative amount of activities required for earning the badge assigned for the relevant contents, except for badge cases dealing with health care (eg, earned 20 likes = 20, 50 links shared = 50, 30 solved problems = 30)

### Badge Design Framework

**Figure 5:**
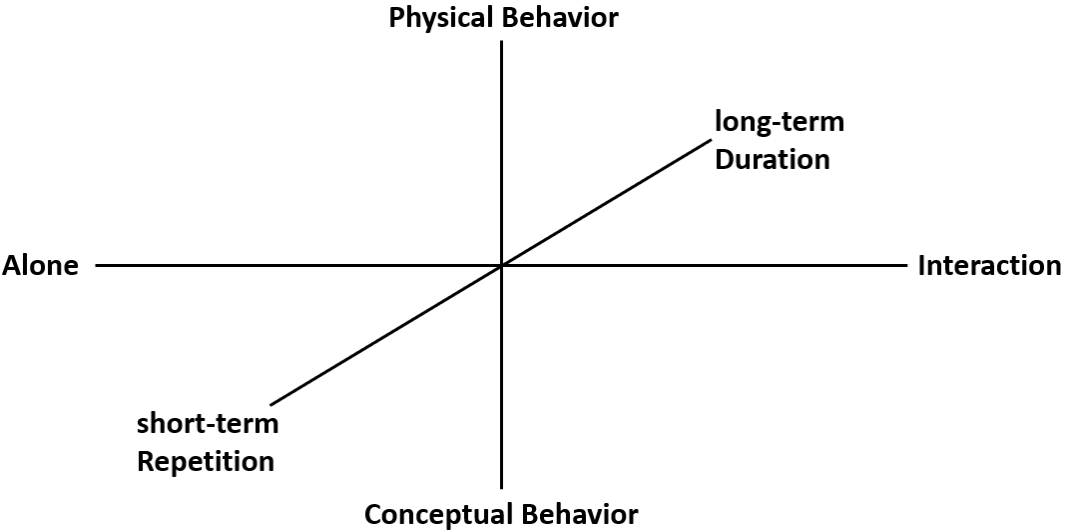
Suggested badge design framework.

Figure 5 illustrates a badge design perspective that applies the MECE approach to suggest a badge design framework. The suggested badge design framework consists of three axes.

The x-axis indicates the interaction of the player participating in an activity. It is divided in terms of individual activity without interaction between players and activity requiring interaction between players. The y-axis indicates the type of learning activity required for earning a badge. This is categorized into the following types: physical and conceptual. The z-axis indicates the time and effort required for investing in a learning activity in order to earn a badge. It is divided as follows: short-term simple repeated activity and long-term complex difficult activity.

#### X-Axis: Interaction Between Players to Earn a Badge—Playing Alone Versus Playing Together

To be precise, carrying out a learning activity alone provides a sense of familiarity. In the gamified learning environment, however, interacting with other learners is effective for improving social skills such as communication [29], listening [30], problem solving, and improving learning motivation [31,32]. In the gamified learning environment, an assignment is provided to the learner in the form of a mission/quest based on the learning content. The missions/quests are categorized based on two criteria: one mission/quest that can be solved by an individual alone and another that encourages two or more learners to interact with each other and solve the problem. The learning activity form is determined based on the theoretical background suggested by the x-axis. To ensure affordance for the learner, the y-axis should determine whether an individual completes a learning activity alone or in cooperation with two or more learners.

Among the cases collected by this research team, Passport to Success [23] required the completion of particular courses and encouraged participation in activities (club activities, volunteering, cardiopulmonary resuscitation training, and local events). Cases from online learning platforms encouraged interaction in a way that commonly allowed learners to share the problem they had solved and receive feedback from one or more people; furthermore, it may evaluate the result of n or more people. Meanwhile, the process of learning a specific skill or knowledge was designed to encourage an individual to learn alone and earn a badge if he or she satisfies certain standards.

#### Y-Axis: The Type of Learning Activity Required for Earning a Badge—Physical Versus Conceptual Activities

According to the experiential learning theory developed by Kolb [33], the methods through which the learner acquires experience during the learning process are categorized based on two types: concrete experience and abstract conceptualization; the learner carries out reflective observation and active experimentation based on such experiences. However, the theory suggests that experiential learning is completed only through cyclical repetitions of the above process. Our study relied on Kolb’s work to suggest appropriate activity types for earning a badge based on concrete experience and abstract conceptualization, which the learner experiences through the learning process. When the teacher designs a learning activity, the relevant learning experiences are provided through physical and conceptual activities. Physical activities can make the learning experience more concrete. Conceptual activities allow the learner to experience abstract conceptualization. Therefore, activity types that can efficiently deliver learning experiences in the learning environment can be divided into two categories: physical and conceptual.

The teacher considers the physical learning environment while providing efficient learning experiences because the physical learning environment is a major variable that affects academic achievement [34]. According to Caldwell [35], learners who completed a learning activity in an ergonomics-based physical learning environment improved their academic achievement by 26.2% compared with learners in other environments. On the other hand, since excessive promotion of physical learning activities in physical learning environments might have a negative effect on the learner [36], it is important to request an appropriate level of physical learning.

Conceptual learning activity plays an important role in the development of reasoning, categorization, memorization, problem solving, and generalization, which cannot be learned through physical activities [37]. The teacher provides the learner with conceptual learning activities and delivers experiences related to creating and using a concept. Using this method can help the learner move beyond simply categorizing objects based on basic rules or features; this helps the learner experience conceptual learning by finding complex rules and new patterns and conceptualizing them. Through this process, the learner experiences abstract concepts and accepts them as a part of their own learning experience.

Among the cases collected by this research team, physical learning activity cases included learning n or more computer programming skills, uploading n or more posts, and posting n or more mentions. Health care apps included walking n steps or running n km. Conceptual activities included solving quizzes related to the learning content, implementing a more effective algorithm (compared with the existing one), uploading n posts, and posting n mentions. As a special case, Khan Academy [20] did not just deliver badges but also introduced great historical figures in related fields (eg, Benjamin Franklin, Frederick Douglass). Among the collected cases, the minimum amount of activities was 3 [20], while the maximum amount of activities was 1000 [19,23]. In the case of health care apps, the activities were walking 41.84 km (26 miles) [19], 72 km (90,000 steps) [19], and 12,861.88 km (7992 miles) [19] and earning 5000 to 2,000,000 points [22]. Passport to Success [23] implemented a condition where a badge was earned when the learner acquired a certain grade point average (GPA) level for a particular subject, grade, or semester.

#### Z-Axis: Time and Effort Invested to Earn a Badge—Simple Repeated Short Term Versus Complex Continued Long Term

Using an experiment, Ebbinghaus [38] proved that knowledge acquired through learning could be forgotten over time. Pedagogy has continued to conduct research in order to solve this problem and thus resolve forgotten knowledge by repeating learning as much as it is forgotten. Dale [39] recommended applying participatory learning methods (group discussion, practice, and teaching others) to the cone of experience instead of passive learning methods (listening to lectures, reading, using audio-visual learning materials, and viewing demonstrations) in order to facilitate efficient learning. This is because learning through interaction with other learners or a teacher is more effective for learning new knowledge and skills compared with sitting alone and struggling with the book. In a gamified learning environment, cognitive apprenticeship [1,40] is established, in which the learner receives the teacher’s knowledge and skill through interaction with the teacher. On a gamified online learning platform, the learner learns basic knowledge and skills from a tutorial and masters them by applying and expanding them while solving a given problem.

The problem of forgetting easier and simpler knowledge and skills can be solved through short-term repeated learning. In a gamified training environment, the learner can acquire knowledge and skills through simple repetitions [41]. However, this approach does not apply to knowledge or skills that are complex and difficult and thus require long-term training. By using game elements, the gamified learning environment provides continuous learning motivation for gaining knowledge or skills that require continuous and long-term training [31]. Therefore, in cases where a gamified learning environment uses a badge, the teacher should encourage the learner to learn easier and simpler knowledge or skills through short-term/repeated learning and set an appropriate period of learning time; furthermore, the teacher should induce a learning activity to help the learner acquire knowledge or skills that require more complex, difficult, and long-term training.

Of the 943 cases collected by this research team, 306 badge cases offered concrete examples. Taking 1 day as 1440 hours, the average period required to earn a badge was 165.46 days, the minimum was 0.01 days (15 minutes) [20], and the maximum was 1460 days (4 years) [19]. For Passport to Success [23], the predetermined period units were quarter, trimester, and semester. Furthermore, when the specific event was held in a local area that used Passport to Success [23], badge gain condition was set to coincide with the event period. Gamified health care apps set this period on a weekly or monthly basis. The period set for earning badges should be established based on the teacher’s experience and knowhow. Academically, there is no equation or theory to calculate the optimal period for earning badges.

## Results

### Suggestions Regarding Badge Types

**Figure 6:**
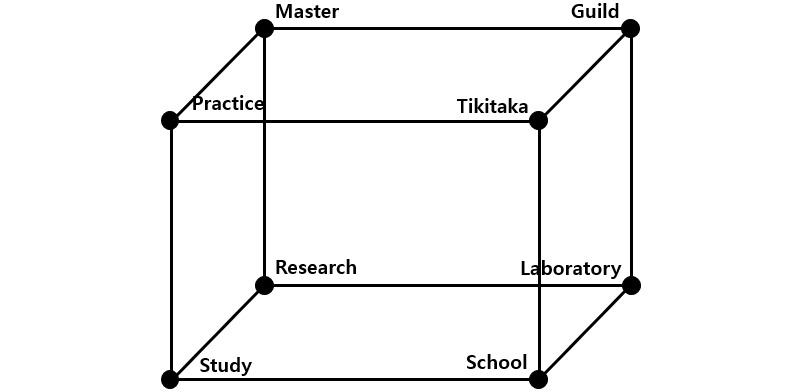
Suggested badge types.

This study suggests eight badge types for three badge design conditions (Figure 6). Table 2 describes the characteristics of each type of badge.

Table 3 shows the analysis results for 943 badges from 11 gamification in education contents that were categorized into this research team’s badge types. We conducted Pearson chi-square tests, and there was a significant difference of chi-square 1117.7, *P*<.001.

Three patterns were extracted from badge cases collected by this research team. First, online platforms were considered. Badges in Codecademy (forum) [13], Codecombat [14], Duolingo [15-17], Edge [18], Khan Academy [20], Memrise (forum) [21], and Sololearn [25] showed a similar type of distribution. It is conjectured that online platforms focused on conceptual activities, since physical activities are limited online. Among these seven online platforms, the proportion of badges that encouraged interactions with other learners instead of individual learning was higher in Codecademy (forum) [13], Memrise (forum) [21], and Sololearn [25]. It is interpreted that these platforms encouraged the learner to share their learning outcome with other learners, receive feedback from them, and thus expand knowledge. Meanwhile, Codecombat [14], Duolingo [15-17], Edge [18], and Khan Academy [20] provided more badges related to individual learning compared with interaction-related ones. It is reckoned that these online platforms intended to encourage the learner to master knowledge over a long-term period through repeated short-term training.

Second, health care apps were also considered. They included Fitbit [19], Nike Plus [22], and Pokemon Go [24]. These contents all commonly feature exercise. They set concrete criteria for encouraging the user’s physical activities, and they increased the distance or the number of necessary steps in order to continue activities. In addition, analysis showed that these health care apps applied badge credentials to the amount of exercise undertaken, which was not very visible; furthermore, they inspired the users to work toward their goals. In the meantime, these contents had a relatively smaller number of badges that encouraged interaction.

Last, Passport to Success [23] was examined. This case was applied to the actual educational setting, and eight types of badges were extracted compared with the other cases. Since it is a learning-related badge, it has been conjectured that its badge distribution is higher among conceptual activities than physical ones. In Passport to Success, however, badges were distributed on a periodic (semester, trimester, and yearly) basis in order to encourage the following physical activities: clubs, bands, and Reserve Officers' Training Corps. For conceptual activities, badges that encouraged the learner to obtain a certain GPA level (B or C, 3.0 or higher, 3.5 or higher, and 4.0 or higher) were arranged on a periodic (semester, trimester, and yearly) basis.

**Table 2:** Description of badge types.

Type	Framework			Description and goal of badge
	X-axis	Y-axis	Z-axis	
Practice	Physical	Alone	Short-term	Badge type for a simpler and easier learning activity that should be repeated alone for a short-term period Badge type that is provided when it is necessary to acquire a lower-level skill in order to learn a higher one
Mastery	Physical	Alone	Long-term	Badge type for a complex and difficult physical learning activity that should be performed alone for a certain period of time Badge type provided when a higher-level skill should be refined by using a lower-level one
Tikitaka	Physical	Interaction	Short-term	Badge type for a simple learning activity that should be repeated with other learners for a short-term period Badge type provided when a basic skill required for performing the final project in a team activity needs to be learned
Guild	Physical	Interaction	Long-term	Badge type for a physical learning activity that requires collaboration and cooperation with other learners for a certain period of time Badge type provided when immersion is needed to produce a final project outcome in a team activity
Study	Conceptual	Alone	Short-term	Badge type provided for an easier and more repetitive conceptual learning activity (eg, memorizing a simple math equation or studying grammar) Badge type used when it is necessary to encourage a basic knowledge learning activity to learn a higher-level concept
Research	Conceptual	Alone	Long-term	Badge type provided for a complex, difficult, and continuous conceptual learning activity (eg, memorizing a calculus equation or learning a difficult algorithm) Badge type provided when immersion and encouragement are required for a learning activity involving a difficult algorithm or a concept based on a lower-level concept
School	Conceptual	Interaction	Short-term	Badge type for a learning activity that encourages collaboration and cooperation in order to overcome limitations at an individual level; it can be acquired over a short-term period Badge type provided for immersion and encouragement in an activity that requires learners to share opinions with one another in order to suggest the final project idea in a team activity
Laboratory	Conceptual	Interaction	Long-term	Badge type provided for a learning activity that encourages continued collaboration and cooperation in order to perform a long-term project or resolve a particular issue Badge type provided when it is necessary to encourage each team member to perform his or her role in order to complete the final team activity project

**Table 3:** The distribution of suggested badge types in collected learning platform cases.

Gamified learning contents	Practice, n (%)	Master, n (%)	Tikitaka, n (%)	Guild, n (%)	Study, n (%)	Research, n (%)	School, n (%)	Laboratory, n (%)
Codecademy (forum)	0 (0)	0 (0)	0 (0)	0 (0)	5 (8)	6 (10)	31 (52)	18 (30)
Codecombat	0 (0)	0 (0)	0 (0)	0 (0)	32 (63)	11 (22)	8 (16)	0 (0)
Duolingo	4 (9)	0 (0)	0 (0)	0 (0)	24 (55)	8 (18)	6 (14)	2 (5)
Edge	0 (0)	0 (0)	0 (0)	0 (0)	4 (17)	2 (8)	1 (4)	17 (71)
Fitbit	29 (47)	33 (53)	0 (0)	0 (0)	0 (0)	0 (0)	0 (0)	0 (0)
Khan Academy	0 (0)	0 (0)	0 (0)	0 (0)	38 (39)	31 (32)	19 (20)	9 (9)
Memrise (forum)	0 (0)	0 (0)	0 (0)	0 (0)	11 (22)	7 (14)	24 (48)	8 (16)
Nike Plus	43 (55)	30 (38)	3 (4)	1 (1)	0 (0)	0 (0)	1 (1)	0 (0)
Passport to Success	6 (2)	13 (4)	17 (6)	12 (4)	120 (40)	57 (19)	44 (15)	31 (10)
Pokemon Go	40 (33)	65 (53)	4 (3)	8 (7)	0 (0)	0 (0)	6 (5)	0 (0)
Sololearn	0 (0)	0 (0)	0 (0)	0 (0)	5 (9)	8 (15)	25 (46)	16 (30)

## Discussion

### Principal Findings

Our study describes a badge design framework for improving the learner’s learning motivation in a gamified learning environment and for introducing innovation into the learning environment. In past educational settings, the badge was simply an extrinsic reward; however, it has now become one of the devices that induces affordance toward self-directed learning by improving learning sustainability, providing learning motivation, and setting goals. Furthermore, badges have begun to be recognized as microcredentials. Mozilla’s Open Badge is one good example that indicates this trend. Open Badges are applied to online learning platforms, and the credentials that the learner has acquired are provided in the form of badges. The provided badge can be indicated on the learner’s social network services such as Facebook and LinkedIn. It is acknowledged as the learner’s acquired badge, even though the learner does not include it in his or her résumé or submit a copy of this credential. As such, the applicability of such badges is expanding gradually.

Therefore, a badge design should be more systematic. In addition to Open Badges, the badge application system provides a badge design tool that can create a badge by inputting an icon, a badge name, a description, and a completion date. However, creating a badge-specific design in order to encourage the learner’s affordance is ultimately up to the teacher or designer.

Devedžić [42] analyzed the advantages of a badge from the perspectives of the learner and the teacher. The learner-centered perspective suggests that badges offer the following benefits: flexibility in the learning environment through the use of badges, voluntary setting of learner goals, visualization of previously completed goals, progress in terms of gaining learning experience, and provision of the possibility to plan and implement a future learning activity. Furthermore, it was revealed that the badge had a positive effect on critical thinking, teamwork, leadership, and abilities or skills/knowledge that had not been recognized properly. Devedžić [42] made suggestions for efficiently reflecting a badge’s characteristics from the perspectives of the learner, the teacher, and the educational institution as follows.

From the learner’s perspective:

- Supporting goal setting, planning, and self-reflection
- Feedback provision through abstraction and integration of learning traces from various learning environments
- Recognition of otherwise underrecognized or nonrecognized skills and prior learning
- Development of a sense of community membership

From the teacher’s perspective:

- Facilitating learners’ motivation and engagement
- Scaffolding the learning process: using badges to chart learning routes for students
- Supporting alternative assessments and feedback provision

From the educational institution’s perspective:

- Improvement of assessment, grading, and feedback collection
- Increased visibility and interschool collaboration and cooperation
- Improvements in instructional and motivational practices

In order to incorporate the above values suggested by Devedžić [29], it is necessary to use the badge design framework developed in this study. Badges designed by using our study’s badge design framework are expected to efficiently deliver the aforementioned seven benefits to the learner. This paper still recommends that users use the existing tool to create a basic badge structure or McIlvenny’s [12] suggested basic badge structure. However, if the three conditions for a badge design framework, as suggested by this research team, are used to encourage the learner’s affordance through badges, it is expected to enable an efficient badge design that can facilitate a learning activity that the teacher wants to conduct.

### Limitations

The following are the limitations and future directions of this study. It is necessary to ensure feasibility by categorizing badges that are actually used in educational settings based on this study’s badge design framework. Since this study developed the framework based on 943 collected cases, it is estimated that it might be difficult to test feasibility based on these collected cases. Therefore, it is necessary to conduct a follow-up study to test this research team’s developed badge design framework. Furthermore, it is necessary to test the effectiveness of badges developed through use of this research team’s badge framework. The basic badge structure can be developed based on previous studies. An additional study should be conducted in order to develop the features that will be included in the badges based on the findings of this study and test whether they are effective in practical terms. To test efficiency in the education environment, it is necessary to conduct a follow-up study by using the existing questionnaire tool. Glynn et al [43] developed the Science Motivation Questionnaire II, which ensured the validity and reliability of this study through statistical tests. The questionnaire tool includes items related to intrinsic motivation, career motivation, self-determination, self-efficacy, and grade motivation. A future study will use this questionnaire tool, design badges using this research team’s developed badge design framework, and analyze their efficiency in actual educational settings.

### Conclusions

Based on our research, we recommend that users design a badge in a way that the eight types of badges are distributed evenly. In the online learning environment, physical learning activities are limited in practical terms. The dual process theory describes a system in which people receive and process information [1]. System 1 uses five senses; alternatively, it can carry out parallel processing automatically and emotionally to acquire new information. System 2 processes new information in a controlled and analytical manner based on a particular set of principles or rules. Usually, people first acquire new knowledge from system 2 and then internalize it in a way that best suits them by using system 1. If physical and conceptual learning activities are balanced based on the previously mentioned information processing mechanisms, the efficiency of learning can be maximized. While it is good to use a badge to learn a particular concept or theory, we suggest that badges should be designed evenly based on our suggested badge design framework; this will help to strike a balance between physical and conceptual activities. Among the badge cases collected by this research team, all the cases (except for Passport to Success) showed a concentrated distribution toward the content’s domain. While there might be a limitation in providing all activities, badges should be designed so that they induce interaction to overcome individual learning limitations, thus encouraging balanced activities.

## Abbreviations

GPA: grade point average
MECE: mutually exclusive collectively exhaustive
